# Case study research and causal inference

**DOI:** 10.1186/s12874-022-01790-8

**Published:** 2022-12-01

**Authors:** Judith Green, Benjamin Hanckel, Mark Petticrew, Sara Paparini, Sara Shaw

**Affiliations:** 1 Wellcome Centre for Cultures & Environments of Health, University of Exeter, Exeter, UK; 2 Institute for Culture and Society, Western Sydney University, Sydney, Australia; 3 Department of Public Health, Environments & Society, London School of Hygiene & Tropical Medicine, London, UK; 4 Wolfson Institute of Population Health, Queen Mary University of London, London, UK; 5 Nuffield Department of Primary Care Health Sciences, University of Oxford, Oxford, UK

**Keywords:** Case study, Evaluation, Causal inference, Inequality, Public health, Health services research

## Abstract

Case study methodology is widely used in health research, but has had a marginal role in evaluative studies, given it is often assumed that case studies offer little for making causal inferences. We undertook a narrative review of examples of case study research from public health and health services evaluations, with a focus on interventions addressing health inequalities. We identified five types of contribution these case studies made to evidence for causal relationships. These contributions relate to: (1) evidence about system actors’ own theories of causality; (2) demonstrative examples of causal relationships; (3) evidence about causal mechanisms; (4) evidence about the conditions under which causal mechanisms operate; and (5) inference about causality in complex systems. Case studies can and do contribute to understanding causal relationships. More transparency in the reporting of case studies would enhance their discoverability, and aid the development of a robust and pluralistic evidence base for public health and health services interventions. To strengthen the contribution that case studies make to that evidence base, researchers could: draw on wider methods from the political and social sciences, in particular on methods for robust analysis; carefully consider what population their case is a case ‘of’; and explicate the rationale used for making causal inferences.

## Background

Case study research is widely used in studies of context in public health and health services research to make sense of implementation and service delivery as enacted across complex systems. A recent meta-narrative review identified four broad, overlapping traditions in this body of work: developing and testing complex interventions; analysing change in organisations; undertaking realist evaluations; and studying complex change naturalistically [1]. Case studies can provide essential thick description of interventions, context and systems; qualitative understanding of the mechanisms of interventions; and evidence of how interventions are adapted in the ‘real’ world [2, 3].

However, in evaluative health research, case study designs remain relegated to a minor, supporting role [4, 5], typically at the bottom of evidence hierarchies. This relegation is largely due to assumptions that they offer little for making the kinds of causal claims that are essential to evaluating the effects of interventions. The strengths of deep, thick studies of specific cases are conventionally set against the benefits of ‘variable-based’ designs, with the former positioned as descriptive, exploratory or illustrative, and the latter as providing the strongest evidence for making causal claims about the links between interventions and outcomes. In conventional hierarchies of evidence, the primary evidence for making causal claims comes from randomised controlled trials (RCTs), in which the linear relationship between a change in one phenomenon and a later change in another can be delineated from other causal factors. The classic account of causality drawn on in epidemiology requires identifying that the relationship between two phenomena is characterised by co-variation; time order; a plausible relationship; and a lack of competing explanations [6]. The theoretical and pragmatic limitations of RCT designs for robust and generalizable evaluation of interventions in complex systems are now well-rehearsed [2, 7–10]. In theory, though, random selection from a population to intervention exposure maximises ability to make causal claims: randomisation minimises risks of confounding, and enables both an unbiased estimate of the effect size of the intervention and extrapolation to the larger population [6]. Guidance for evaluations in which the intervention cannot be manipulated, such as in natural experiments, therefore typically focuses on methods for addressing threats to validity from non-random allocation in order to strengthen the credibility of probabilistic causal effect estimates [4, 11].

This is, however, not the only kind of causal logic. Case study research typically draws on other logics for understanding causation and making causal inferences. We illustrate some of the contributions made by case studies, drawing on a narrative review of research relating to one particularly enduring and complex problem: inequalities in health. The causal chains linking interventions to equity outcomes are long and complex, with recognised limitations in the evidence base for ‘what works’ [12]. Case study research, we argue, has a critical role to play in making claims about whether, how and why interventions reduce, mitigate, or exacerbate inequalities. Our examples are drawn from a broader review of case study research [1] and supporting literature reviews [5], from which we focused on cases which had an explanatory aim, and which shed light on how interventions in public health or health services might reduce, create or sustain inequality. In this paper, we: i) outline some different *kinds* of evidence relevant to causal relationships that can be  derived from case study research; ii) outline what is needed for case study research to contribute to explanatory, as well as exploratory claims; and iii) advocate for greater clarity in reporting case study research to foster discoverability.

### Cases and causes

There are considerable challenges in defining case study designs or approaches in ways that adequately delineate them from other research designs. Yin [13], for instance, one of the most highly cited source texts on case studies in health research [1], resists providing a definition, instead suggesting case study research is more a strategy for doing empirical research. Gerring [14] defines case study research as: “*an intensive study of a single unit for the purpose of understanding a larger class of (similar) units*” (p342, emphasis in original). This definition is useful in suggesting the basis for the inferences drawn from cases, and the need to consider the relationships between the ‘case’ (and phenomena observed within it) and the population from which it is drawn. Gerring notes that studies of single cases may have a greater “affinity” for descriptive aims, but that they can furnish “evidence for causal propositions” ( [14], p347). Case studies are, he suggests, more likely to be useful in elucidating deterministic causes: those conditions that are necessary and/or sufficient for an outcome, whereas variable based designs have advantages for demonstrating probabilistic causation, where the aim is to estimate the likelihood of two phenomena being causally related. Case studies provide evidence for the mechanisms of causal relationships (e.g. through process tracing, through observing two variables interacting in the real world) and corroboration of causal relationships (for instance, through pattern matching).

Gerring’s argument, drawing on political science examples, is that there is nothing epistemologically distinct about research using the case study: rather, it has particular affinities with certain styles of causal modelling. We take this as a point of departure to consider not *whether* case studies can furnish evidence to help with causal inference in health research, but rather *how* they have done this. From our examples on case study research on inequalities in health, we identify the *kinds* of claims that relate to causality that were made. We note that some relate to (1) *Actors’ accounts of causality*: that is, the theories of those studied about if, how and why interventions work. Other types of claim use various kinds of comparative analytic logic to elucidate evidence of causal relationships between phenomena. These claims include: (2) *Demonstrations of causal relationships* – in which evidence from one case is sufficient for identifying a plausible causal relationship; (3) *Mechanisms* – evidence of the mechanisms through which causal relationships work; (4) *Conditions*—evidence of the conditions under which such mechanisms operate; and (5) *Complex causality*—evidence for outcomes that arise from complex causality within a system. This list is neither mutually exclusive, nor exhaustive: many case studies aim to do several of these (and some more). It is also a pragmatic rather than theoretical list, focusing on the kinds of evidence claimed by researchers rather than the formal methodological underpinnings of causal claims (for a discussion of the latter, see Rohlfing [15]).

## What kinds of causal evidence do case studies provide?

### Actors’ accounts of causality

This is perhaps the most common kind of evidence provided by case study research. Case studies, through in-depth research on the actors within systems, can generate evidence about how those actors themselves account for causal relationships between interventions and outcomes. This is an overt aim of many realist evaluation studies, which focus on real forces or processes that exist in the world that can provide insight into causal mechanisms for change.

Ford and colleagues [16], for example, used a series of five case studies of local health systems to explore socio-economic inequalities in unplanned hospital admission. Cases were selected on the basis of either narrowing or widening inequalities in admission, with a realist evaluation focused on delineating the context-mechanisms-outcome (CMO) configurations in each setting, to develop a broader theory of change for addressing inequalities. The case study approach used a mix of methods, including drawing on documentary data to assess the credibility of mechanisms proposed by health providers. The authors identified 17 distinct CMO configurations; and five factors that were related to trends for inequalities in emergency admissions, including health service factors (primary care workforce challenges, case finding and proactive case management) and those external to the health service (e.g., financial constraints on public services, residential gentrification). Ford and colleagues noted that none of the CMO configurations were clearly associated with improved or worsening trends in inequalities in admission.

Clearly, actors’ accounts of causality are not in themselves evidence of causality. Ford and colleagues noted that they interrogated accounts for plausibility (e.g. that interventions mentioned were prior to effects claimed) and triangulated these accounts with other sources of data, but that inability to empirically corroborate the hypothesized CMO links limited their ability to make claims about causal inference. This is crucial: actors in a system may be aware of the forces and processes shaping change but unaware of counterfactuals, and they are unlikely to have any privileged insight into whether factors are causal or simply co-occurring (see, for instance, Milton et. al. [17] on how commonly cited ‘barriers’ in accounts of not doing evaluations are also evident in actors’ accounts of doing successful evaluations). Over-interpretation of qualitative accounts of insiders’ claims about causal relationships as if they provide conclusive evidence of causal relationships is poor methodology.

This does not mean that actors’ accounts are not of value. First, in realist evaluation, as in Ford and colleagues’ study [16], these accounts provide the initial theories of change for thinking about the potential causal pathways in logic models of interventions. Second, insiders’ accounts of causality are part of the system that is being explained. An example comes from Mead and colleagues [18], who used a case study drawing largely on qualitative interviews to explore “how local actors from public health, and the wider workforce, make sense of and work on social inequalities in health” ( [18] p168). This used a case study of a partnership in northwest England to address an enduring challenge in inequalities policy: the tendency for policies that address upstream health determinants to transform, in practice, to focus more on behavioural and individual level factors**.** Local public health actors in the partnership recognised the structural causes of unequal health outcomes, yet discourses of policy action tended to focus only on the downstream, more individualising levels of health, and on personal choice and agency as targets for intervention. Professionals conceptualised action on inequality as relating only to the health of the poorest, rather than as a problem of a gradient in health outcomes across society. There was a geographical localism in their approach, which framed particular places as constellations of health and social problems. Drawing on theory from figurational sociology, Mead and colleagues note that actors’ own accounts are the starting point of an analysis, which then puts those accounts into play with theory about how such discourses are reproduced. The researchers suggest that partnership working itself exacerbated the individualising frameworks used to orient action, as it became a hegemonic framing, reducing the possibilities for partnerships to transform health inequalities. Here, then, a case study approach is used to shed light on the causes of a common failure in policies addressing inequalities. The authors take seriously the divergence of actors’ own accounts of causality and those of other sources, and analyse these as part of the system.

Finally, insider accounts should be taken seriously as contributing to evidence about causal inference through shedding light on the complex looping effects of theoretical models of causality and public accounts. For instance, Smith and Anderson [19], drawing on a meta-ethnographic literature review of ‘lay theorising’ about health inequalities, note that, counter to common assumptions, public understanding of the structural causes of health inequalities is sophisticated: but that it may be disavowed to avoid stigma and shame and to reassert some agency. This is an important finding for informing knowledge exchange, suggesting that further ‘awareness raising’ may be unnecessary for policy change, and counter-productive in needlessly increasing stigma and shame.

### Demonstrations of causal relationships

When strategically sampled, and rooted in a sound theoretical framework, studies of single cases can provide evidence for generalizable causal inferences. The strongest examples are perhaps those that operate as ‘black swans’ for deterministic claims, in that one case may be all that is needed to show that a commonly held assumption is not generalizable. That is, a case study can demonstrate unequivocally that one phenomenon is not inevitably related to another. These can come from cases sampled because they are extreme or unusual. Prior’s [20] study of a single man in a psychiatric institution in Northern Ireland, for instance, showed that, counter to Goffman’s [21] original theory of how ‘total institutions’ lead to stigmatisation and depersonalisation, the effects of institutionalisation depended on context—in this case, how the institution related to the local community and the availability of alternative sources of self-worth available to residents.

Strategically sampled typical cases can also provide demonstrative evidence of causal relationships. To take the enduring health services challenge of inequalities in self-referral to emergency care, Hudgins and Rising’s [22] case study of a single patient is used to debunk a common assumption that high use of emergency care is related to inappropriate care-seeking by low-income patients. They look in detail at the case of “a 51-year-old low-income, recently insured, African American man in Philadelphia (USA) who had two recent ED [emergency department] visits for evaluation of frequent headaches and described fear of being at risk for a stroke.” ( [22] p50). Drawing on theories of structural violence and patient subjectivity, they use this single case to shed light on why emergency department use may appear inappropriate to providers. They analyse the interplay of gender roles, employment, and insurance status in generating competing drivers of health seeking, and point to the ways in which current policies deterring self-referral do not align well with micro- and macro-level determinants of service use. The study authors also note that because their methods generate data on ‘why’ as well ‘what’ people do, they can “lay the groundwork” ( [22], p54] for developing future interventions. Here, again, a single case is sufficient. In understanding the causal pathways that led to this patient’s use of emergency care, it is clear why policies addressing inequalities through deterring low-income users would be unlikely to work.

### Mechanisms: how causal relationships operate

A strength of case study approaches compared with variable-based designs is furnishing evidence of how causal relationships operate, deriving from both direct observations of causal processes and from analysis of comparisons within and between cases. All cases contain multiple observations; variations can be observed over time and space, across or within cases [14]. Observing regularities, co-variation and deviant or surprising findings, and then using processes of analytic induction [23] or abductive logic [24] to derive, develop and test causal theories using observations from the case, can build a picture of causal pathways.

Process tracing is one formal qualitative methodology for doing this. Widely used in political and policy studies, but less in health evaluations [25], process tracing links outcomes with their causes, focusing on the mechanisms that link events on causal pathways, and on the strength of evidence for making connections on that causal chain. This requires sound theoretical knowledge (such that credible hypotheses can be developed), well described cases (ideally at different time points), observed causal processes (the activities that transfer causes to effects), and careful assessment of evidence against tests of varying strength for the necessity and sufficiency for accepting or rejecting a candidate hypothesis [26, 27]. In health policy, process tracing methods have been combined to good effect with quantitative measures to examine casual processes leading to outcomes of interest. Campbell et. al. [28], for instance, used process tracing to look at four case studies of countries that had made progress towards universal health coverage (measured through routine data on maternal and neonatal health indicators), to identify key causal factors related to health care workforce.

An example of the use of process tracing in evaluation comes from Lohmann and colleagues’ [25] case study of a single country, Burkina Faso, to examine why performance based financing (PBF) fails to improve equity. PBF, coupled with interventions to improve health care take up among the poor, aims to improve health equity in low and middle-income countries, yet impact evaluations suggest that these benefits are typically not realised. This case study drew on data from the quantitative impact assessment; programme documentation; the intervention process evaluation; and primary qualitative research for the process tracing, in the light of the theory of change of the intervention. Lohmann and colleagues [25] identified that a number of conditions that would have been necessary for the intervention to work had not been met (such as eligible patients not receiving the card needed to access health care or providers not receiving timely reimbursement). A key finding was that although implementation challenges were a partial cause of policy failure, other causal conditions were external to the intervention, such as lack of attention to the non-health care costs incurred by the poorest to access care. Again, a single case, if there are good grounds for extrapolating to similar contexts (i.e., those in which transport is required to access health care), is enough to demonstrate a necessary part of the causal pathway between PBF and intended equity outcomes.

### Conditions under which causal mechanisms operate

The example of ‘transport access’ as a necessary condition for PBF interventions to ‘work’ also illustrates a fourth type of causal evidence: that relating to the transferability of interventions. Transferable causal claims are essential for useful evidence: “(f)or policy and practice we do not need to know ‘it works somewhere’. We need evidence for ‘it-will-work-for-us’ claims: the treatment will produce the desired outcome in our situation as implemented there” ( [8] p1401). Some causal mechanisms operate widely (using a parachute will reduce injury from jumping from a plane; taking aspirin will relieve pain); others less so. In the context of health services and public health research, few interventions are likely to be widely generalizable, as the mechanisms will operate differently across contexts [7]. This context dependency is at the heart of realist evaluations, with the assumption that underlying causal mechanisms require particular contexts in order to operate, hence the focus on ‘how, where, and for whom’ interventions work [29]. Making useful claims therefore requires other kinds of evidence, relating to what Cartwright and Munro [30] call the ‘capacities’ of the intervention: what power it has to work reliably, what stops it working, what other conditions are needed for it to work. This evidence is critical for assessing whether an intervention is likely to work in a given context and to assess the intended and unintended consequences of intervention adoption and implementation. Cartwright and Munro’s recommendation is therefore to study causal powers rather than causes. That is, as well as interrogating whether the intervention ‘causes’ a particular outcome, it is also necessary to address the potential for and stability of that causal effect. To do that entails addressing a broader range of questions about the causal relationship, such as how the intervention operates in order to bring about changes in outcomes; what other conditions need to be present; what might constrain this effect; what other factors within the system also promote or constrain those effects; and what happens when different capacities interact? [30]. Case study research can be vital in providing this kind of evidence on the capacities of interventions [31].

One example is from Gibson and colleagues [32], who use within-case comparisons to shed light on why a ‘social prescribing’ intervention may have different effects across socioeconomic classes. These interventions, typically entailing link workers who connect people with complex health care needs to local services and resources, are often framed as a way to address enduring health inequalities. Drawing on sociological theory on how social class is reproduced through socially structured and unequal distribution of resources (‘capitals’), and through how these shape people’s practices and dispositions, Gibson and colleagues [32] explicate how capitals and dispositions shaped encounters with the intervention. Their analysis of similarities and differences within their case (of different clients) in the context of theory enables them to abstract inferences from the case. Drawing out the ways in which more advantaged clients mobilised capital in their pursuit of health, with dispositions more closely aligned to the intervention, they unravel classed differences in ability to benefit from the intervention, with less advantaged clients inevitably having ‘shorter horizons’ focused on day to day challenges: “This challenges the claim that social prescribing can reduce inequalities, instead suggesting it has the potential to exacerbate existing inequalities” ( [32], p6).

Case studies can shed light on the capacities of interventions to improve or exacerbate inequalities, including identifying unforeseen consequences. Hanckel and colleagues [33, 34], for example, used a case study approach to explore implementation of a physical health intervention involving whole classes of children running for 15 min each day in the playground in schools in south London, UK. This documented considerable adaption of the intervention at the level of school, class and pupil, and identified different pathways through which the intervention might impact on inequalities. In terms of access, the intervention appeared to be equitable, in that there was no evidence of disproportionate roll out to schools with more affluent pupils or to those with fewer minority ethnic pupils [33]. However, identifying the ‘capacities’ of the intervention also identified other pathways through which it could have negative equity effects. The authors found that in practice, the intervention emphasised body weight rather than physical activity, and intervention roll-out reinforced class and ethnicity-based stigmatising discourses about lower income neighbourhoods [34].

### Complex causality

There is increasing recognition that the systems that reproduce unequal health outcomes are complex: that is, that they consist of multiple interacting components that cannot be studied in isolation, and that change is likely to be non-linear, characterised by, for instance, phase shifts or feedback loops [35]. This has two rather different implications. First, case study designs can be particularly beneficial for taking a system perspective on interventions. Case studies enable a focus on aspects that are not well explicated through other designs, such as how context interacts with interventions within systems [7], or on how multiple conditional pathways might link interventions and outcomes [36]. Second, when causation is not linear, but ‘emergent’, in that it is not reducible to the accumulated changes at lower levels, evaluation designs focused on only one outcome at one level (such as weight loss in individuals) may fail to identify important effects. Case studies have an invaluable role here in unpacking and surfacing these effects at different levels within the systems within which interventions and services are delivered. One example is transport systems, which have been the focus of considerable public health interest to encourage more ‘active’ modes, in which more of the population walk or cycle, and fewer drive. However, more simplistic evaluations looking at one part of a causal chain (such as that between traffic calming interventions and local mode shift) may fail to appreciate how systems are dynamic, and that causation might be emergent. This is evident in a case study of transport policy impacts from Sheller [37], who takes the case of Philadelphia, USA, to reveal how this post-car trend has racialized effects that can exacerbate inequality. Weaving in data from participant observations, historical documentary sources and statistical evidence of declining car use, Sheller documents the racialized impacts of transport policies which may have reduced car use and encouraged active modes overall, but which have largely prioritised ‘young white’ mobility in the context of local gentrification and neglect of public transit.

One approach to synthesising evidence from multiple case studies to make claims about complex causation is Qualitative Comparative Analysis (QCA), which combines quantitative methods (based on Boolean algebra) with detailed qualitative understanding of a small to medium N sample of cases. This has strengths for identifying multiple pathways to outcomes, asymmetrical sets of conditions which lead to success or failure, or ‘conjunctural causation’, whereby some conditions are only causally linked to outcomes in relation to others [38]. There is growing interest in using these approaches in evaluative health studies [39]. One example relating to the effectiveness of interventions addressing inequalities in health comes from Blackman and colleagues [36], who explored configurations of conditions which did or did not lead to narrowing inequalities in teenage conception rates across a series of local areas as cases. This identified some surprising findings, including that ‘basic’ rather than good or exemplary standards of commissioning were associated with narrowing the equity gap, and that the proportion of minority ethnic people in the population was a key condition.

## Discussion

Not all case study research aims to contribute to causal inference, and neither should it [1, 5, 40]. However, it can. We have identified five ways in which case study evidence has contributed to causal explanations in relation to a particularly intractable challenge: inequalities in health. It is therefore time to stop claiming that case study designs have only a supporting role to play in evaluative health research. To develop a theoretical evidence base on ‘what works’, and how, in health services and public health, particularly around complex issues such as addressing unequal health outcomes, we need to draw on a greater range of evidential resources for informing decisions than is currently used. Best explanations are unlikely to be made from single studies based on one kind of causality, but instead will demand some kind of evidential pluralism [41]. That is, one single study, of any design, is unlikely to generate evidence for all links in complex causal chains between an intervention and health outcomes. We need a bricolage of evidence from a diverse range of designs [42] to make robust and credible cases for what will improve health and health equity. This will include evidence from case studies, both from single and small N studies, and from syntheses of findings from multiple cases.

Our focus on case studies that shed light on interventions for health inequalities identified the critical role that case studies can play in theorising, illuminating and making sense of: system actors’ own causal reasoning; whether there are causal links between intervention and outcome; what mechanism(s) might link them; when, where and for whom these causal relationships operate; and how unequal outcomes can be generated from the operation of complex systems. These examples draw on a range of different theoretical and methodological approaches, often from the wider political and social sciences. The approaches illustrated are rooted in very different, even incompatible, philosophical traditions: what researchers understand by ‘causality’ is diverse [43]. However, there are two commonalities across this diversity that suggest some conditions for producing good case studies that can generate evidence to support causal inferences. The first is the need for theoretically informed and comparative analysis. As Gerring [14] notes, causal inferences rely on comparisons – across units or time within a case, or between cases. It is comparison that drives the ability to make claims about the potential of interventions to produce change in outcomes of interest, and under what conditions. There are a range of approaches to qualitative data analysis, and choice of method has to be appropriate for the kinds of causal logics being explicated, and the availability of data on particular phenomena within the case. Typically, though, this will require analysis that goes beyond descriptive thematic analysis [31]. Approaches such as process tracing or analytic induction require both fine-grained and rigorous comparative analysis, and a sound theoretical underpinning that provides a framework for making credible inferences about the relationships between phenomena within the case and to the wider population from which the case is selected.

This leads to the second commonality: the need to clarify what the case is a case ‘of’, and how it relates to other candidate cases. What constitutes a ‘case’ is inevitably study specific. The examples we have drawn on include: PBF in a country [25], transport systems in a city [37], and a social prescribing intervention in primary care [32]. Clearly, in other contexts, each of these ‘cases’ could be sampling units within variable based studies (of financing systems, or countries; of infrastructures systems, or cities in a state; of particular kinds of service intervention, or primary care systems). Conversely, these cases could be populations within which lower level phenomena (districts, neighbourhoods, patients) are studied. What leads to appropriate generalisations about causal claims is a sound theorisation of the similarities and particularities of the case compared with other candidate cases: how Burkina Faso has commonalities with, or differences from, other settings in which PBF has failed to improve equity; or the contexts of gentrification and residential churn that make Philadelphia similar to other cities in the US; or the ways in which class-based dispositions and practices intersect with similar types of service provisions.

A critical question remains: How can well-conducted case study evidence be better integrated into the evidence base? Calls for greater recognition for case study designs within health research are hardly new: Flyvberg’s advocacy for a greater role for case studies in the social sciences [44] has now been cited around 20,000 times, and calls for methodological pluralism in health research go back decades [42, 45, 46]. Yet, case studies remain somewhat neglected, with ongoing misconceptions about their limited role, despite calls for evidence based medicine to incorporate evidence for mechanisms as complementary to evidence of correlation, rather than as inferior [47]. Even where the value of case studies for contributing to causal inference is recognised, searching for good evidence is not straightforward. Case studies are neither consistently defined nor necessarily well reported. Some of the examples in this paper do not use the term ‘case study’ in the title or abstract, although they meet our definition. Conversely, many small scale qualitative studies describe themselves as ‘case studies’, but focus on thick description rather than generalisability, and are not aiming to contribute to evaluative evidence. It is therefore challenging, currently, to undertake a more systematic review of empirical material. Forthcoming guidance on reporting case studies of context in complex systems aims to aid discoverability and transparency of reporting (Shaw S, et al: TRIPLE C Reporting Principles for Case study evaluations of the role of Context in Complex interventions, under review). This recommends including ‘case study’ in the title, clarifying how terms are used, and explicating the philosophical base of the study. To further advance the usefulness of case study evidence, we suggest that where an aim is to contribute to causal explanations, researchers should, in addition, specify their rationales for making causal inferences, and identify what broader class of phenomena their case is a case ‘of’.

## Conclusions

Case study research can and does contribute to evidence for causal inferences. On challenging issues such as addressing health inequalities, we have shown how case studies provide more than detailed description of context or process. Contributions include: describing actors’ accounts of causal relationships; demonstrating theoretically plausible causal relationships; identifying mechanisms which link cause and effect; identifying the conditions under which causal relationships hold; and researching complex causation.

## Data Availability

Not applicable; no new data generated in this study. For the purpose of open access, the author has applied a ‘Creative Commons Attribution (CC BY) licence to any Author Accepted Manuscript version arising.

## Abbreviations

CMO: Context-mechanism-outcome
PBF: Performance based financing
QCA: Qualitative Comparative Analysis
RCT: Randomised controlled trial
